# Discrete Unilateral Constrained Extended Kalman Filter in an Embedded System

**DOI:** 10.3390/s25154636

**Published:** 2025-07-26

**Authors:** Leonardo Herrera, Rodrigo Méndez-Ramírez

**Affiliations:** 1 Independent Researcher, Monterey, CA 93943, USA; 2 Paradigm Advanced Research Center (PARC), 5340 Canotek Rd., Unit #4, Ottawa, ON K1J9C6, Canada; rmendez@paradigm.com

**Keywords:** unilateral constraint, Extended Kalman Filter, embedded system, dsPIC, microcontroller, digital-to-analog converter

## Abstract

Since its publication in the 1960s, the Kalman Filter (KF) has been a powerful tool in optimal state estimation. However, the KF and most of its variants have mainly focused on the state estimation of smooth systems. In this work, we propose a new algorithm called the Discrete Unilateral Constrained Extended Kalman Filter (DUCEKF) that expands the capabilities of the Extended Kalman Filter (EKF) to a class of hybrid mechanical systems known as systems with unilateral constraints. Such systems are non-smooth in position and discontinuous in velocity. Lyapunov stability theory is invoked to establish sufficient conditions for the estimation error stability of the proposed algorithm. A comparison of the proposed algorithm with the EKF is conducted in simulation through a case study to demonstrate the superiority of the DUCEKF for the state estimation tasks in this class of systems. Simulations and an experiment were developed in this case study to validate the performance of the proposed algorithm. The experiment was conducted using electronic hardware that consists of an Embedded System (ES) called “Mikromedia for dsPIC33EP” and an external DAC-12 Click board, which includes a Digital-to-Analog Converter (DAC) from Texas Instruments.

## 1. Introduction

The Kalman Filter (KF) [1], proposed in the 1960s, is an optimal state estimator that has been used in many applications. Over the years, its capabilities have been expanded to generalize to a wide range of systems and scenarios. Although most of these generalizations are focused on smooth systems, there have been a few works on non-smooth systems, e.g., [2,3,4,5,6,7,8].

This work aims to expand the capabilities of the EKF to a class of hybrid mechanical systems known as systems with unilateral constraints. In such a system, the unilateral constraint restricts the system’s motion in only one direction, which prevents penetration between the system and an impact surface while still allowing for impacts to occur. As an example, a ball resting on a table is a system under unilateral constraints because it cannot move downward through the table but is free to move in other directions. These systems are characterized by differential and difference equations with a switching rule that defines the commutation between these equations. Such systems are non-smooth in position and discontinuous in velocity at the impact surface, where the discrete dynamic comes into play. These systems have received much attention, especially in control engineering [9,10,11,12,13]. Examples of them include the walking biped robot [14], Newton’s pendulum [15], the pendulum with barrier [12], and the Bouncing Ball [16].

This paper builds upon [17] to meet real-world applications typically conceived in digital implementations. In this paper, the differential equation of the system is discretized for digital implementation. As a result, two different equations and a switching rule define the system with unilateral constraints treated in this research. The difference equation derived from the differential equation describes the dynamics beyond the impact surface, while the other equation defines the dynamics at this surface. The resulting system remains non-smooth in position and discontinuous in velocity in the presence of impacts.

The DUCEKF is introduced for the state estimation in such a system. The DUCEKF consists of the discrete EKF, a discrete transition, and a switching rule. The EKF performs estimations beyond impacts in the smooth branch. On the other side, the discrete transition is responsible for estimations at the impact surface, where the non-smooth branch occurs. Sufficient conditions for the stability of the proposed algorithm are derived based on Lyapunov stability theory. Since the DUCEKF is defined for discrete systems, the employed Lyapunov theory is discrete. This differs from the continuous treatment used in [17] and ensures stability within this discrete framework, which is essential for embedded implementation.

A Lyapunov function depending on the DUCEKF Difference Riccati Equation (DRE) solution and estimation error is defined for the non-smooth branch. The function guides the online modification of the DRE solution, which ensures that the Lyapunov function does not grow after impacts. The modified DRE solution and the estimations at the non-smooth branch constitute the DUCEKF at this phase and are employed to reinitialize the EKF to take over again at the smooth branch. Stability is a significant contribution, as in previous studies on non-smooth KF-based estimations, the stability of the estimation error is not often focused [2,3,4,6,8]. However, some works, such as [5,7], do emphasize Lyapunov stability and robustness.

In Matlab, the DUCEKF is numerically exemplified with a case study, the Discrete Hybrid Van der Pol Oscillator (DHVPO), a discrete version of the Hybrid Van der Pol Oscillator from [18]. The DUCEKF is also compared with the EKF in the case study to show the advantages of the DUCEKF over the EKF in the state estimation task of systems with unilateral constraints. The superiority of DUCEKF over EKF is evident by looking at the behavior of the Lyapunov function, which shows stability for the DUCEKF estimations while showing instability for the EKF estimations.

After simulations in MATLAB 2024b, the algorithm is implemented in the ES “Mikromedia for dsPIC33EP” to meet real-world applications. The ES is powered by a 16-bit dsPIC33EP512MU810 microcontroller and features the Thin Film Transistor (TFT) Liquid Crystal Display (LCD) module for multimedia applications, where the graphical results on the real and estimated phase portraits are displayed for comparison.

As the algorithm was initially developed in MATLAB, the algorithm was ported to the C programming language using the MikroC PRO for dsPIC. The code was developed for implementation on the Mikromedia dsPIC33EP board. This compact and efficient development platform enables real-time user interaction and visual monitoring of system behavior [19].

An external DAC-12 Click board was integrated via SPI with the ES to enhance the system’s functionality. This board includes the DAC60508, an eight-channel DAC from Texas Instruments, which generates analog output signals corresponding to the estimated and real states. These signals are displayed on an oscilloscope as a time series for comparison of real vs. estimated. C code was also developed in the ES for DAC integration.

The ES implementation proves that the proposed DUCEKF can be implemented in an ES, as the EKF [20,21]. ES implementation is a significant contribution, as embedded systems are the means to real-world applications such as the automotive industry, medical services, the Internet of Things, security systems, etc.

The proposed algorithm is well-suited for sensor data fusion and state estimation in systems operating under unilateral constraints. It advances standard sensor data fusion techniques, primarily based on KF and its variants, which are designed for smooth systems. Additionally, this algorithm can function as a state estimator for unmeasured states, thereby reducing the cost or complexity associated with sensors.

The organization of the document is as follows: the preliminaries are in Section 2, the DUCEKF in Section 3, the electronic implementation in Section 4, the conclusions in Section 5, and an appendix detailing the algorithm implementation is in Appendix A.

## 2. Preliminaries

For later use, the discrete EKF, the discrete system with unilateral constraints, and the description of the DHVPO employed to exemplify the proposed approach are detailed.

### 2.1. Discrete Extended Kalman Filter

Consider the nonlinear systems described by the difference equation

(1) $$x_{k}=f\left(x_{k-1}\right)+w_{k},$$

where $x_{k-1}={\left[x_{k-1}{\left(1\right)}^{⊤}\;\;x_{k-1}{\left(2\right)}^{⊤}\right]}^{⊤}$ is the state vector, $f\left(x_{k-1}\right)={\left[f_{1}{\left(x_{k-1}\right)}^{⊤}\;\;f_{2}{\left(x_{k-1}\right)}^{⊤}\right]}^{⊤}$ a nonlinear function that defines the dynamics of the system, and $w_{k}$ is a vector of White Gaussian Noise (WGN) normally distributed as $N(0,Q_{k})$, with 0 mean and covariance matrix $Q_{k}$. For (1), the output is

(2) $$y_{k}=h\left(x_{k}\right)+v_{k},$$

where $h(x_{k})$ is a vector function of the state vector, and $v_{k}$ is the vector of WGN normally distributed as $N(0,R_{k})$, where 0 is the mean and $R_{k}$ is the covariance matrix of $v_{k}$.

The EKF estimates the state vector of (1) from the combination of the output (2) and the model of (1).

The EKF can be found in the literature [22], and its description is as follows:

(3) $$\begin{array}{ccc} {\hat{x}}_{k}^{p} & = & f({\hat{x}}_{k-1}^{u}), \end{array}$$

(4) $$\begin{array}{ccc} P_{k}^{p} & = & A_{k-1}P_{k-1}^{u}A_{k-1}^{⊤}+Q_{k}, \end{array}$$

(5) $$\begin{array}{ccc} K_{k} & = & P_{k}^{p}C_{k}^{⊤}{\left(C_{k}P_{k}^{p}C_{k}^{⊤}+R_{k}\right)}^{-1}, \end{array}$$

(6) $$\begin{array}{ccc} {\hat{x}}_{k}^{u} & = & {\hat{x}}_{k}^{p}+K_{k}\left(y_{k}-h\left({\hat{x}}_{k}^{p}\right)\right), \end{array}$$

(7) $$\begin{array}{ccc} P_{k}^{u} & = & \left(I-K_{k}C_{k}\right)P_{k}^{p}. \end{array}$$

Description of the variables in the EKF are given below:

- ${\hat{x}}_{k}^{p}={\left[{\hat{x}}_{k}^{p}{\left(1\right)}^{⊤}\;\;{\hat{x}}_{k}^{p}{\left(2\right)}^{⊤}\right]}^{⊤}$ is the estimation of the state $x_{k}$ before $y_{k}$.
- $P_{k}^{p}$ is the predicted covariance matrix of the estimation error.
- $K_{k}$ is the Kalman gain.
- ${\hat{x}}_{k}^{u}={\left[{\hat{x}}_{k}^{u}{\left(1\right)}^{⊤}\;\;{\hat{x}}_{k}^{u}{\left(2\right)}^{⊤}\right]}^{⊤}$ describes, although locally, the optimal estimation of $x_{k}$.
- $P_{k}^{u}$ is the updated covariance matrix of the estimation error.
- $Q_{k}$ and $R_{k}$ are symmetric and positive definite matrices capturing in their diagonals the variances of $w_{k}$ and $v_{k}$, respectively.

The superindexes *p* and *u* indicate the prediction and update phases of the EKF. The matrices

$$\begin{array}{c} A_{k-1}=\frac{\partial f(x_{k-1})}{\partial x_{k-1}}{|}_{{\hat{x}}_{k-1}^{u}},\;\;C_{k}=\frac{\partial h(x_{k})}{\partial x_{k}}{|}_{{\hat{x}}_{k}^{p}} \end{array}$$

result from the linear approximations of (1) and (2) around the nominal values $x_{k-1}={\hat{x}}_{k-1}^{u}$, $x_{k}={\hat{x}}_{k}^{p}$, $w_{k}=0$, and $v_{k}=0$. These approximations are

(8) $$\begin{array}{ccc} x_{k} & \approx & A_{k-1}x_{k-1}+u_{k-1}+w_{k}\;\;\mathrm{and} \end{array}$$

(9) $$\begin{array}{ccc} y_{k} & \approx & h\left({\hat{x}}_{k}^{p}\right)+C_{k}x_{k}-C_{k}{\hat{x}}_{k}^{p}+v_{k}, \end{array}$$

with $u_{k-1}=f\left({\hat{x}}_{k-1}^{u}\right)-A_{k-1}{\hat{x}}_{k-1}^{u}$. The idealized initial conditions for the EKF are ${\hat{x}}_{0}^{u}=E\left[x_{0}\right]$ and $P_{0}^{u}=$ var$\left[x_{0}\right]$, where *E* and var are, respectively, the expectation and variance operators.

### 2.2. Discrete System with Unilateral Constraints

Given the unilateral constraint $F(x_{k}\left(1\right))\geq \varepsilon$, the discrete system with unilateral constraints is described by (10).

(10) $$\mathrm{System}=\left\{\begin{array}{cc} \mathrm{Smooth}\;\mathrm{branch}\;\left(1)–(2\right), & F(x_{k}\left(1)\right)>\varepsilon \\ \mathrm{Non}–\mathrm{smooth}\;\mathrm{branch}\;(11), & F(x_{i-1}\left(1\right))=\varepsilon \end{array}\right.$$

Equations (1) and (2) hold beyond the impact surface $F(x_{i-1}\left(1\right))=\varepsilon$, and (11) takes over at this surface when an impact occurs at the discrete time i−1. The function $\mu \left(x_{i-1}\right)={\left[x_{i-1}{\left(1\right)}^{⊤}\;\;\mu_{1}{\left(x_{i-1}\right)}^{⊤}\right]}^{⊤}$ defines a constant position and discontinuous velocity in this surface, $x_{i}={\left[x_{i}{\left(1\right)}^{⊤}\;\;x_{i}{\left(2\right)}^{⊤}\right]}^{⊤}$ is the state vector at this point, and $\varepsilon =[\varepsilon_{0},0)$ is an activation set that defines a range for all the $x_{i-1}$ events, with $\varepsilon_{0}>0$.

(11) $$x_{i}=\mu \left(x_{i-1}\right).$$

### 2.3. Discrete Hybrid Van der Pol Oscillator

A case study system that will be used later to exemplify our contribution is the DHVPO, a discrete version of the oscillator from [18]. Such an oscillator can produce a discontinuous limit cycle that bifurcates into an asymptotically stable origin under certain parameters. Its solution has served as a reference in the control of mechanical systems [12,23]. Its discrete version forms the case study system that follows the generic definition (10). Given the unilateral constraint $\underset{F(x_{k}\left(1\right))}{\underset{︸}{x_{k}\left(1\right)}}\geq \varepsilon$, the oscillator is described by (12).

(12) $$\mathrm{DHVPO}=\left\{\begin{array}{cc} \mathrm{Continuous}\;\mathrm{Van}\;\mathrm{der}\;\mathrm{Pol}\;\left(13)–(14\right), & x_{k}\left(1\right)>\varepsilon \\ \mathrm{Transition}\;(15), & x_{i-1}\left(1\right)=\varepsilon ,x_{k}\left(2\right)<0 \end{array}\right.$$

Beyond the impact surface, when $x_{k}\left(1\right)>\varepsilon$, the oscillator is governed by (13) and (14). On the other side, it is described by (15) when the impact surface is reached at $x_{i-1}\left(1\right)=\varepsilon$ and $x_{k}\left(2\right)<0$. In addition to the position constraint $x_{i-1}\left(1\right)=\varepsilon$, the velocity constraint $x_{k}\left(2\right)<0$ is considered to activate the non-smooth phase in this case study.

(13) $$\underset{x_{k}}{\underset{︸}{\left[\begin{array}{c} x_{k}\left(1\right)\\ x_{k}\left(2\right) \end{array}\right]}}=\underset{f(x_{k-1})}{\underset{︸}{\left[\begin{array}{c} x_{k-1}\left(1\right)\\ x_{k-1}\left(2\right) \end{array}\right]+\left[\begin{array}{c} x_{k-1}\left(2\right)\\ -\alpha \left[\left(x_{k-1}{\left(1\right)}^{2}+\frac{x_{k-1}{\left(2\right)}^{2}}{\mu^{2}}\right)-\rho^{2}\right]x_{k-1}\left(2\right)-\mu^{2}x_{k-1}\left(1\right) \end{array}\right]\Delta T}}+\underset{w_{k}}{\underset{︸}{\left[\begin{array}{c} w_{k}\left(1\right)\\ w_{k}\left(2\right) \end{array}\right]}}$$

(14) $$y_{k}=\underset{h(x_{k})}{\underset{︸}{x_{k}\left(1\right)}}+v_{k}$$

(15) $$\underset{x_{i}}{\underset{︸}{\left[\begin{array}{c} x_{i}\left(1\right)\\ x_{i}\left(2\right) \end{array}\right]}}=\underset{\mu (x_{i-1})}{\underset{︸}{\left[\begin{array}{c} x_{i-1}\left(1\right)\\ -\kappa x_{i-1}\left(2\right) \end{array}\right]}}$$

In the transition (15), i=1,2,⋯ are impact instants that occur when the oscillator hits the impact surface. According to [18], a discontinuous limit cycle is generated when the bifurcation parameters α=μ=1, ρ=1.5, and κ=0.5 are defined. The Euler method was employed to get the discrete version (13). Assuming $x_{k}\left(1\right)$ and $x_{k}\left(2\right)$ as the position and velocity of a mechanical system, only position measurements are considered to be available, i.e., $h\left(x_{k}\right)=x_{k}\left(1\right)$ in (2).

## 3. Discrete Unilateral Constrained Extended Kalman Filter

Given the unilateral constraint $F({\hat{x}}_{k}^{u}\left(1\right))\geq \varepsilon$, the proposed filter is defined by (16). Such a filter is constituted by the discrete EKF (3)–(7), the transition (17)–(18), and the switching rule defined within (16).

(16) $$\mathrm{DUCEKF}=\left\{\begin{array}{cc} \mathrm{EKF}\;\left(3)–(7\right), & F({\hat{x}}_{k}^{u}\left(1\right))>\varepsilon \\ \mathrm{Transition}\;\left(17)–(18\right), & F({\hat{x}}_{i-1}^{u}\left(1\right))=\varepsilon \end{array}\right.$$

Beyond the impact surface, when $F({\hat{x}}_{k}^{u}\left(1\right))>\varepsilon$, the DUCEFK is the discrete EKF. However, when the surface is reached at $F({\hat{x}}_{i-1}^{u}\left(1\right))=\varepsilon$, the DUCEFK is defined by the transition (17)–(18).

(17) $${\hat{x}}_{i}^{u}=\mu \left({\hat{x}}_{i-1}^{u}\right)$$

(18) $$P_{i}^{u}=\Omega P_{i-1}^{u}$$

Equation (17) defines a constant estimated position and discontinuous estimated velocity, where ${\hat{x}}_{i}^{u}={\left[{\hat{x}}_{i}^{u}{\left(1\right)}^{⊤}\;\;{\hat{x}}_{i}^{u}{\left(2\right)}^{⊤}\right]}^{⊤}$ and $\mu \left({\hat{x}}_{i-1}^{u}\right)={\left[{\hat{x}}_{i-1}^{u}{\left(1\right)}^{⊤}\;\;\mu_{1}{\left({\hat{x}}_{i-1}^{u}\right)}^{⊤}\right]}^{⊤}$. Equation (18) defines the propagation of the updated covariance matrix, and sufficient conditions are derived in the next theorem, right after the assumption, for this to guarantee asymptotic stability in the Lyapunov sense.

**Assumption** **1.** *Assume that the system and the DUCEKF trajectories reach the impact surfaces $F(x_{i-1}\left(1\right))=\varepsilon$ and $F({\hat{x}}_{i-1}^{u}\left(1\right))=\varepsilon$ simultaneously, then the estimation error at this surface is*

(19) $$e_{i}=\mu \left(e_{i-1}+{\hat{x}}_{i-1}^{u}\right))-\mu \left({\hat{x}}_{i-1}^{u}\right),$$

*where $e_{i}={\left[e_{i}{\left(1\right)}^{⊤}\;\;e_{i}{\left(2\right)}^{⊤}\right]}^{⊤}={\left[x_{i}{\left(1\right)}^{⊤}-{\hat{x}}_{i}^{u}{\left(1\right)}^{⊤}\;\;x_{i}{\left(2\right)}^{⊤}-{\hat{x}}_{i}^{u}{\left(2\right)}^{⊤}\right]}^{⊤}$.*

**Theorem** **1.** *Provided that the previous assumption is satisfied, the propagation of the covariance defined by (18) establishes sufficient conditions for the Lyapunov stability of the estimation error (19).*

**Proof.**  Considere the Lyapunov inequality

(20) $$V\left(e_{i}\right)-V\left(e_{i-1}\right)\leq 0$$

expressed as a function of the DRE solution and the estimation error

(21) $$\begin{array}{c} V\left(\delta e_{i}\right)-V\left(\delta e_{i-1}\right)=\delta e_{i}^{⊤}P_{i}^{u}\delta e_{i}\\ -\delta e_{i-1}^{⊤}P_{i-1}^{u}\delta e_{i-1}\leq 0. \end{array}$$

In (21), $\delta e_{i}$ is the local post-impact estimation error, coming from the following linearization

(22) $$\delta e_{i}=\tilde{A}\delta e_{i-1}$$

of (19) around the nominal value $e_{i-1}=0$, where

(23) $$\tilde{A}=\frac{\partial \left[\mu \left(e_{i-1}+{\hat{x}}_{i-1}^{u}\right)\right)-\mu \left({\hat{x}}_{i-1}^{u}\right)]}{\partial e_{i-1}}{|}_{e_{i-1}=0}.$$

Finally (21) can be written as

(24) $$\begin{array}{c} V\left(\delta e_{i}\right)-V\left(\delta e_{i-1}\right)=\\ \delta e_{i-1}^{⊤}\left[{\tilde{A}}^{⊤}P_{i}^{u}\tilde{A}-P_{i-1}^{u}\right]\delta e_{i-1}\leq 0, \end{array}$$

where the definition (18), with an appropriate selection of the gain Ω forces the inequality (24) to be satisfied. Therefore Lyapunov stability is proved. □

The covariance propagation (18) with an appropriate selection of Ω will compensate for any unstable effects produced by the state transition (17). The updated estimate ${\hat{x}}_{i}^{u}$ and covariance $P_{i}^{u}$ from (17) and (18) are then utilized to reinitialize the EKF to take over again beyond the impact surface, in the smooth branch. According to the theorem, the gain Ω is derived from a systematic approach based on Lyapunov stability analysis. The gain is a key component of the DUCEKF, which is used for state estimation in the generic class of systems defined by Equation (10). The propagation of the covariance matrix is based on the gain to fulfill Lyapunov stability. An explicit solution for this gain can be obtained from Equation (24). In the following section, a case study comparison will demonstrate the significance of this gain; stability will be observed when it is applied, while instability will be evident when it is not.

In addition to the estimation error (19) defined under the assumption, two more definitions of estimation error are feasible. These are shown below.

- The system trajectory reaches the impact surface during the time instants i=1,2,⋯ and the estimated trajectory remains beyond this surface, i.e., $F(e_{i-1}\left(1\right)+{\hat{x}}_{i-1}^{u}\left(1\right))=\varepsilon$ and $F({\hat{x}}_{k}^{u}\left(1\right))\neq \varepsilon$. For this scenario, the error is
  (25) $$e_{i}=\mu \left(e_{i-1}+{\hat{x}}_{i-1}^{u}\right)-{\hat{x}}_{k}^{u}$$
  where ${\hat{x}}_{k}^{u}$ is the estimated state still beyond the impact surface. The time indices *i* and *k* are employed to denote the state at this surface and beyond, respectively. However, they occur at the same time.
- The estimated trajectory reaches the constraint during the impact time instants i=1,2,⋯ and the system trajectory remains beyond this surface, i.e., $F({\hat{x}}_{i-1}^{u}\left(1\right))=\varepsilon$ and $F(e_{k}\left(1\right)+{\hat{x}}_{k}^{u}\left(1\right))\neq \varepsilon$. For this scenario, the error is
  (26) $$e_{i}=e_{k}+{\hat{x}}_{k}^{u}-\mu \left({\hat{x}}_{i-1}^{u}\right)$$
  where $e_{k}+{\hat{x}}_{k}^{u}$ is the system’s state still beyond the impact surface. Again, the time indexes *i* and *k* are employed to denote state at this surface and beyond, respectively. However, they occur at the same time.

To keep the assumption, the DUCEKF is synchronized online by the trajectory of the system, i.e., the unilateral constraint $F({\hat{x}}_{i}^{u}\left(1\right))\geq 0$ of the DUCEKF is replaced by that of the system, $F(x_{i}\left(1\right))\geq 0$, to produce a reset event depending on the system. The DUCEKF at the impact surface is then described by (17) and (18), provided that $F(x_{i-1}\left(1\right))=\varepsilon$ is satisfied.

If the system’s trajectory intersects with the impact surface before the estimated trajectory does, (11) and (17)–(18) are activated for both trajectories, regardless of the estimated trajectory’s location. However, if the estimated trajectory reaches the impact surface first, it will remain there, waiting for the system to make contact. Once the system hits the surface, (11) and (17)–(18) take place.

As per synchronization at the impact surface, the state of the system is assumed to be known at this point. This assumption is valid because a sensor that measures impacts, such as a force sensor, can be integrated into real systems.

## 4. Results

This section presents the results of the DUCEKF (16) performance for the case study DHVPO (12). Both numerical results obtained from MATLAB simulations and experimental results from the electronic hardware are included. The parameters and initial conditions used for this case study are listed in Table 1. The parameter Ω is defined as in (28) to fulfill the Lyapunov stability expressed in (24), turning (24) into (29). Equations (27) and (30) provide an explicit definition for the parameters *A* and $\tilde{A}$.

(27) $$A=\left[\begin{array}{cc} 1 & \Delta T\\ (-2\alpha {\hat{x}}_{k-1}^{u}\left(1\right){\hat{x}}_{k-1}^{u}\left(2\right)-\mu^{2})\Delta T & 1+(-\alpha {\hat{x}}_{k-1}^{u}{\left(1\right)}^{2}-\frac{3\alpha {\hat{x}}_{k-1}^{u}{\left(2\right)}^{2}}{\mu^{2}}+\alpha \rho^{2})\Delta T \end{array}\right].$$

(28) $$\Omega =\left[\begin{array}{cc} 1 & -\frac{1}{\kappa }\\ -\frac{1}{\kappa } & \frac{1}{\kappa^{2}} \end{array}\right].$$

(29) $$\begin{array}{c} V\left(\delta e_{i}\right)-V\left(\delta e_{i-1}\right)=0. \end{array}$$

(30) $$\tilde{A}=\left[\begin{array}{cc} 1 & 0\\ 0 & -\kappa \end{array}\right].$$

### 4.1. MATLAB Simulation

**Figure 1:**
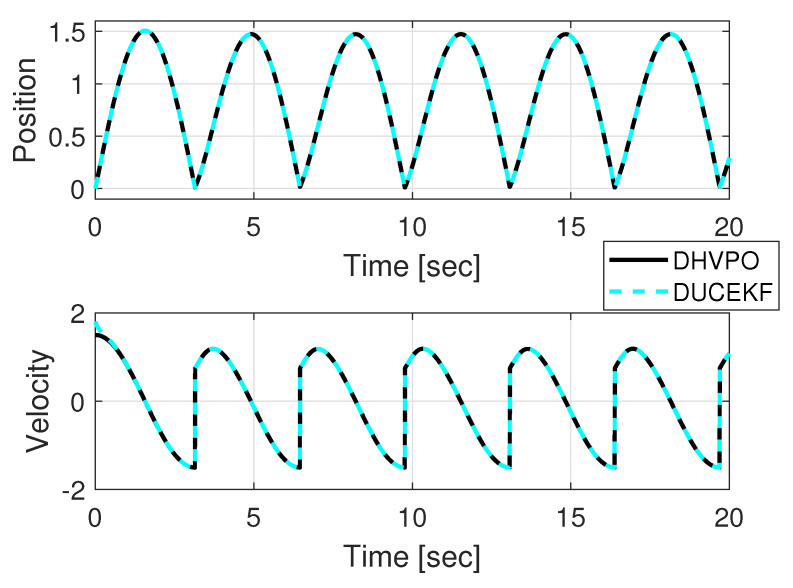
Estimated position and velocity from DUCEKF converge to the DHVPO position and velocity, even when starting from different initial conditions.

**Figure 2:**
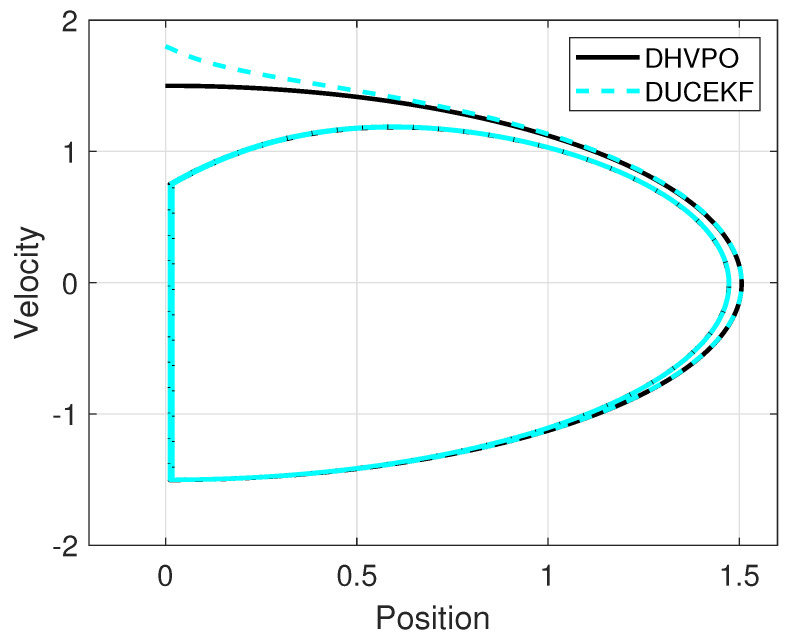
Estimated phase portrait from DUCEKF converges to the DHVPO phase portrait, even when starting from different initial conditions.

Simulation results from MATLAB are in Figure 1 and Figure 2. Figure 1 shows the time evolution of the estimated and the real states for comparison, whereas Figure 2 shows the phase portraits of the estimated and real states for spatial comparison. Despite different initial conditions, the estimates converge to the real state.

A comparison between the DUCEKF and the EKF is conducted in the numerical setting to demonstrate the advantages of the former over the latter in the case study. To ensure a fair comparison, the constraint (17) is integrated into the EKF at the impact surface. The difference between the two methods lies in the covariance update (18), which is only part of the DUCEKF but not of the EKF. For this comparison, the parameter κ=1.5 is utilized to create an unstable estimation error transition (19) and to showcase the capabilities of the DUCEKF over the EKF for the unstable scenario.

**Figure 3:**
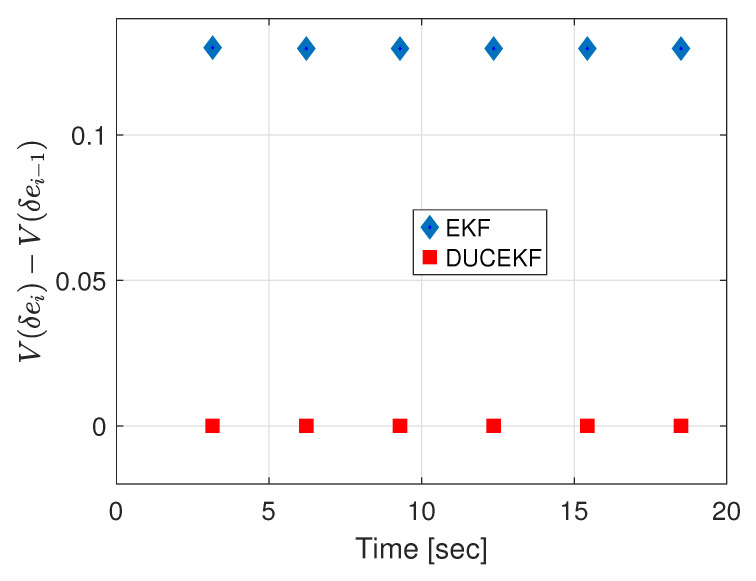
Comparison of Lyapunov inequalities for EKF and DUCEKF shows that the Lyapunov inequality for DUCEKF confirms stability, while the Lyapunov inequality for EKF indicates instability.

The advantages of DUCEKF over the EKF are shown by the behavior of the Lyapunov function at the impact surface. For the DUCEKF, the Lyapunov function shows stability by remaining at zero. The function shows stability even for unstable estimation error transitions, thanks to the compensation provided by the covariance update (18). In contrast, the Lyapunov function for the EKF shows instability by remaining positive on the impact surface. The comparison of the Lyapunov functions for DUCEKF and EKF is shown in Figure 3.

### 4.2. Experimental Results

**Figure 4:**
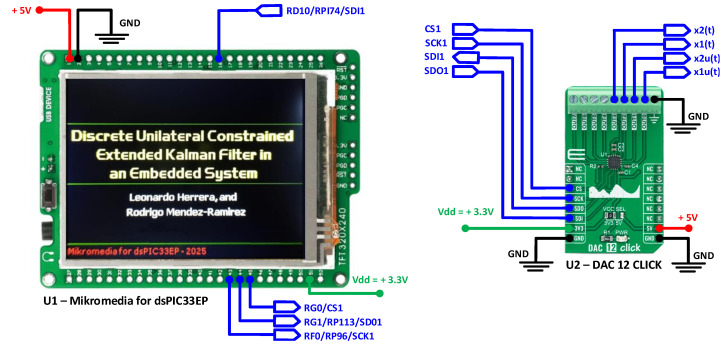
Hardware used in the electronic implementation. On the left, the U1-Mikromedia for dsPIC33EP. On the right, U2-DAC 12 Click board.

The hardware setup utilized for implementation is shown in Figure 4. It includes the Mikromedia for dsPIC33EP (U1) and the DAC-12-Click board (U2). U1 features a TFT LCD screen that is used to display phase-plane portraits, whereas U2 features a precise 12-bit DAC60508 from Texas Instruments to output analog states.

In this experiment, the case study DHVPO is not an external hardware system; rather, it consists of state-space discrete equations implemented in the dsPIC within U1. It is implemented in dsPIC along with DUCEKF and additional code subroutines for data transmission to the DAC and TFT-LCD.

The main algorithm, consisting of DHVPO and DUCEKF, was first programmed in MATLAB and then ported to C using MikroC PRO for dsPIC for real-time execution in U1 and U2. Customized functions were developed in C for all matrix-related calculations, such as matrix multiplication, addition, subtraction, and inversion. The functions were aimed to minimize execution time and computational load in addition to their main objective. Communication between U1 and U2 was established using the SPI protocol, with U1 acting as the master and U2 as the slave. The SPI bus control on U1 was configured to operate in 8-bit mode. To complete a data write operation, three 8-bit sequences are required to control U2, resulting in a 24-bit (3-byte) transmission from master U1 to slave U2. The first 8 bits in the transmission define the channel number in the DAC, and the next 16 bits contain the 12 bits of data for the specific state variable, with the remaining 4 bits padded to complete the 24-bit transmission. The transmission was carried out for each of the four channels assigned to the states $x_{k}\left(1\right),x_{k}\left(2\right),{\hat{x}}_{k}^{u}\left(1\right),{\hat{x}}_{k}^{u}\left(2\right)$ generated by the DHVPO and DUCEKF, respectively.

The experimental execution time of the algorithm took 68.3 ms per iteration and is detailed below in three processes:

- In the first process, the estimated average execution time of the main algorithm on the dsPIC33EP512MU810 in U1 was 248 µs per iteration. The execution time is based on the clock frequency and code efficiency, which was aimed at minimizing the execution time of operations.
- In the second process, the four state variables $x_{k}\left(1\right),x_{k}\left(2\right),{\hat{x}}_{k}^{u}\left(1\right),{\hat{x}}_{k}^{u}\left(2\right)$ were reproduced by DAC60508 in 120 µs per iteration.
- In the third process, a graphic routine generated points and lines to display the phase portraits on the TFT-LCD screen of U1. This process took 67.932 ms per iteration to convert the numerical data into a text string and display it, illustrating estimated and real phase portraits.

The execution time for the three processes is 68.3 ms per iteration. This indicates that the ILI9341 TFT-LCD graphic controller updates a full frame every 68.3 ms under our design conditions, corresponding to a refresh rate of about 14.6 frames per second. The execution time of 68.3 ms is sufficient to reliably display data on the TFT-LCD, ensuring clear and real-time visualization for the end user. A shorter execution time may result in unintelligible data, compromising the clarity of the visual output. The appendix at the end of the document gives a detailed implementation.

**Figure 5:**
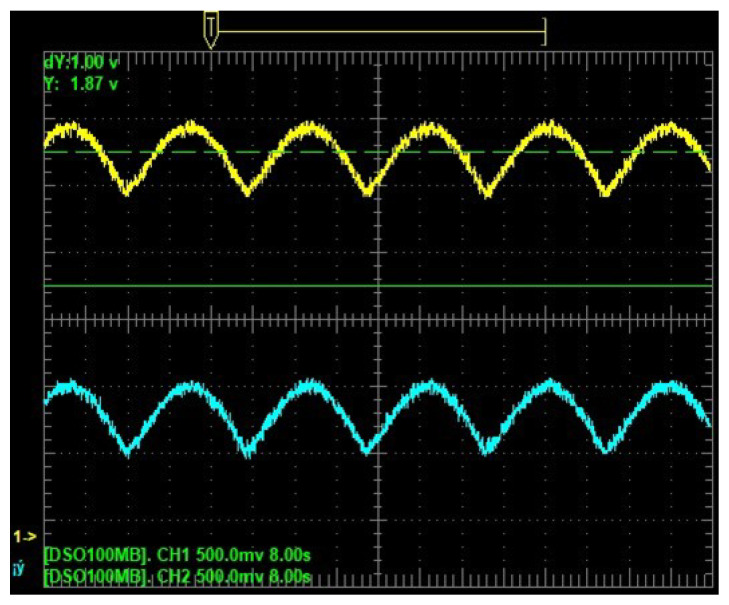
Estimated position from DUCEKF in cyan and real from DHVPO in yellow.

**Figure 6:**
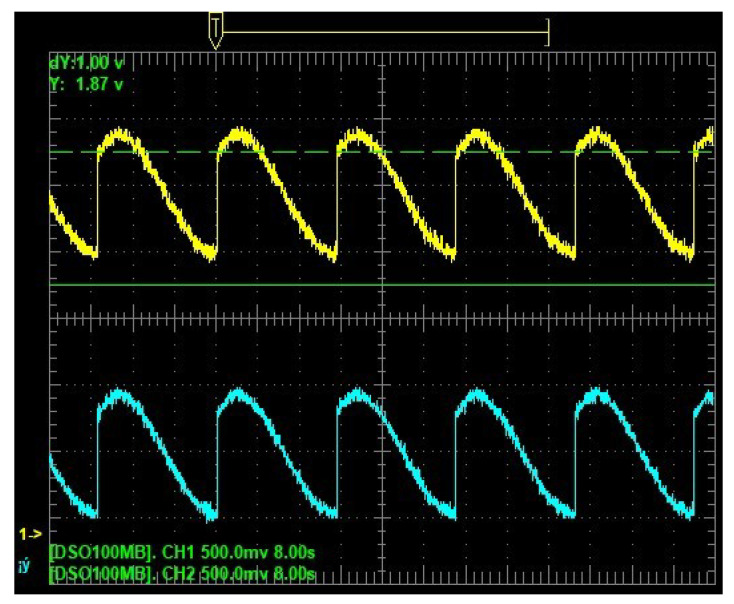
Estimated velocity from DUCEKF in cyan and real from DHVPO in yellow.

**Figure 7:**
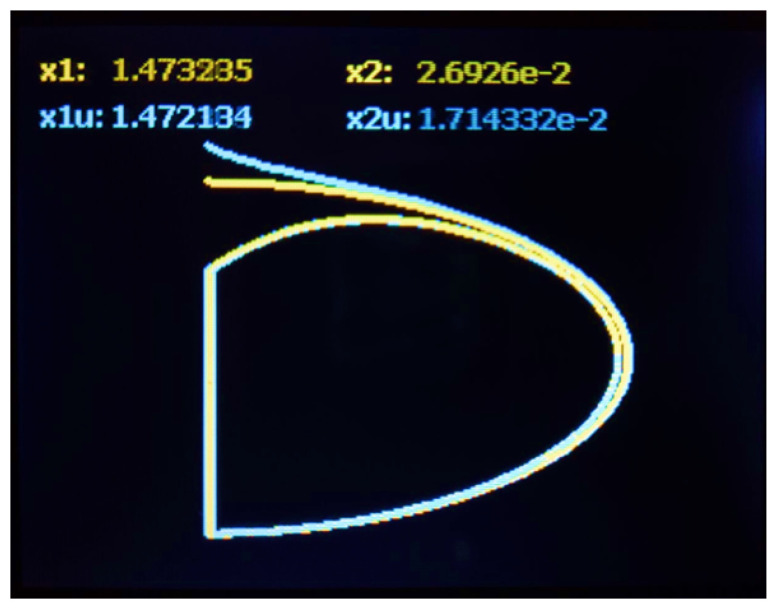
Comparison of discrete phase portraits in TFT LCD screen. Estimated portrait from DUCEKF in cyan and real from DHVPO in yellow. On top of figure the current state numeric values for estimation and real.

Experimental results from hardware are in Figure 5, Figure 6 and Figure 7. Figure 5 shows the time evolution of the position, whereas Figure 6 shows the velocity. The estimated position and velocity exhibit similar amplitude and phase compared with the real ones. The data shown in Figure 5 and Figure 6 are analog outputs produced by the Texas Instruments DAC60508 after being converted from digital to analog. Such data were measured using an oscilloscope and displayed on its screen. Figure 7 shows a comparison of the estimated and real phase portraits displayed on the TFT LCD module of the ES. Tracking of DUCEKF is also confirmed in the phase portraits, despite different initialization.

## 5. Conclusions

The DUCEKF algorithm was introduced for state estimation in mechanical systems with unilateral constraints. Lyapunov stability theory was utilized to derive sufficient conditions for the algorithm’s estimation error stability. These conditions involved an online modification of the DRE solution in the presence of impacts to ensure Lyapunov stability in this phase. Numerical comparison showed the superiority of the DUCEKF over the EKF in the state estimation task of systems with unilateral constraints. Numerical results also confirmed a successful tracking of the DUCEKF estimated state to the state of the DHVPO, validating the algorithm’s effectiveness and motivating implementation in ES as per its discrete description. Experimental results on the ES integrated with the Texas Instrument DAC also showed a successful tracking of the DUCEKF estimated state to the state of the DHVPO. Experimental performance was shown in both discrete phase portraits displayed in the TFT LCD module and analog time series displayed on the oscilloscope screen. The proposed algorithm and its implementation encourage its application in the automation of various relevant autonomous systems, such as humanoid robots or bipedal walking robots, due to its compact design, affordability, and ability to provide real-time state estimation.

## Figures and Tables

**Table 1:** Parameters and initial conditions.

Parameters	Values	Initial Conditions	Values
$Q_{k}$	$0.001I_{2\times 2}$	${\hat{x}}_{0}^{u}\left(1\right)$	0
$R_{k}$	0.0001	${\hat{x}}_{0}^{u}\left(2\right)$	1.8
ε	0.02	$x_{0}\left(1\right)$	0
*C*	[10]	$x_{0}\left(2\right)$	1.5
*A*	Equation (27)	-	-
Ω	Equation (28)	$P_{0}^{u}$	$0.2I_{2\times 2}$
$\tilde{A}$	Equation (30)	-	-
ΔT	0.01	-	-
$w_{k},\;v_{k}$	0	-	-

**Figure A1:**
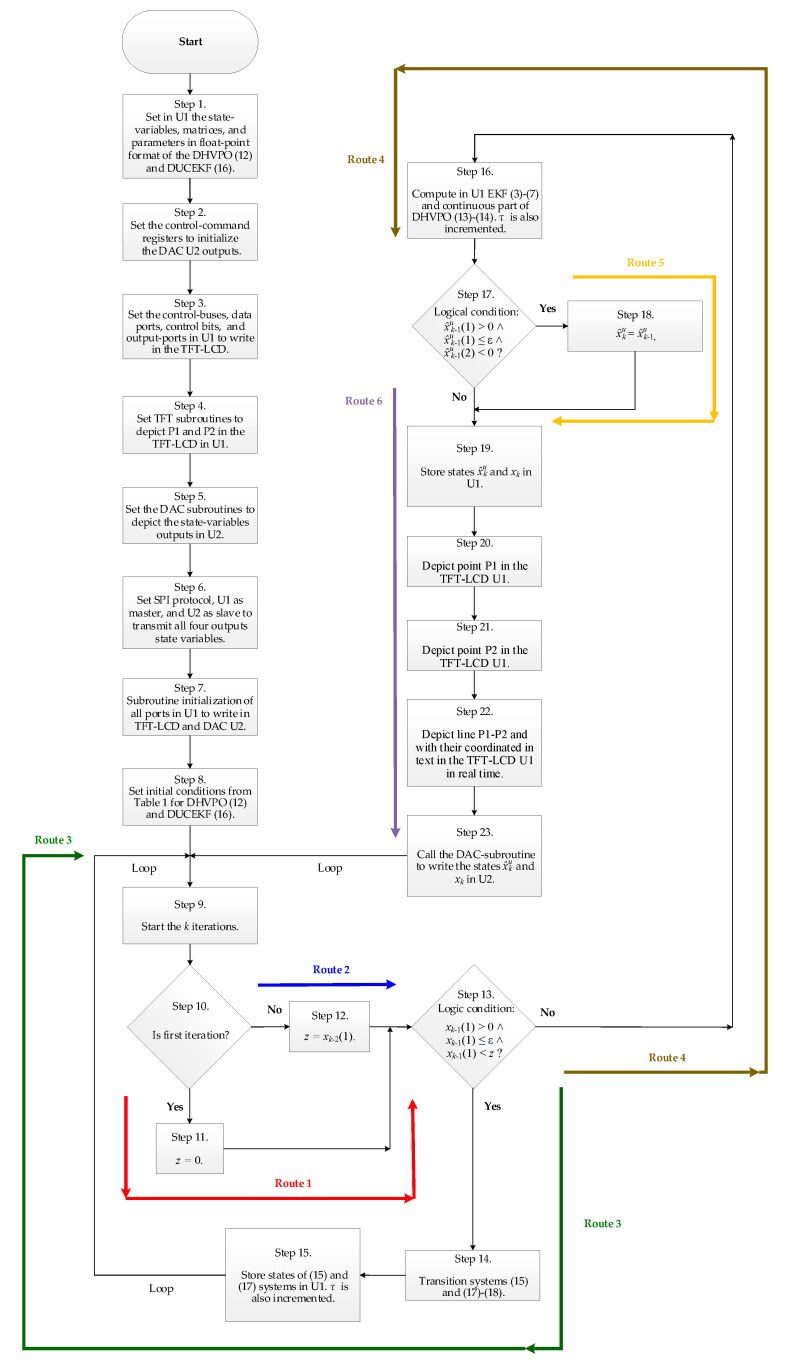
Flowchart depicting the 23 steps required for implementing DHVPO (12) and DUCEKF (16) systems on the embedded system.

## Data Availability

Data available upon request to the corresponding author.

## Abbreviations

The following abbreviations are used in this manuscript:

DAC	Digital-to-Analog Converter
DHVPO	Discrete Hybrid Van der Pol Oscillator
DRE	Difference Riccati Equation
DUCEKF	Discrete Unilateral Constrained Extended Kalman Filter
EKF	Extended Kalman Filter
ES	Embedded System
KF	Kalman Filter
LCD	Liquid Crystal Display
SPI	Serial Peripheral Interface
TFT	Thin Film Transistor
WGN	White Gaussian Noise

## Appendix A. Algorithm Implementation

Section 4.2 provided a general overview of the three key processes involved in the implementation. In this appendix, we provide a detailed explanation of the algorithm’s operation, including timing analysis, statistical path analysis, and a comprehensive review of the entire implementation of the DHVPO (12) and DUCEKF (16) within the embedded system. The algorithm, consisting of 23 steps, was developed and implemented to compute and display the phase-plane trajectories of the DHVPO (12) and DUCEKF (16) systems on a TFT-LCD integrated within U1, while simultaneously outputting the states $x_{k}\left(1\right),x_{k}\left(2\right),{\hat{x}}_{k}^{u}\left(1\right),{\hat{x}}_{k}^{u}\left(2\right)$ through DAC U2. Figure A1 presents the flowchart depicting the 23 steps required for implementing (12) and (16) on the embedded system.

Table A1 summarizes the execution times of the three key processes, which will be explained in detail in the following subsection that also describes the 23 steps of the main flowchart.

**Table A1:** Process description and timing for DHVPO (12) and DUCEKF (16) algorithm execution.

Process	Description	Step	Time (μs)
1	Main algorithm of DHVPO (12) and DUCEKF (16) computed on U1.	9–19	248
2	Graphical representation of the phase plane $x_{k}\left(1\right)-x_{k}\left(2\right)$ from DHVPO (12) versus phase plane ${\hat{x}}_{k}^{u}\left(1\right)-{\hat{x}}_{k}^{u}\left(2\right)$ from DUCEKF (16). Numerical values of the states $x_{k}\left(1\right)$, $x_{k}\left(2\right)$, ${\hat{x}}_{k}^{u}\left(1\right)$, and ${\hat{x}}_{k}^{u}\left(2\right)$ are also displayed on the TFT-LCD.	20–22	67,932
3	Output of state variables $x_{k}\left(1\right)$ and $x_{k}\left(2\right)$ of DHVPO (12), and ${\hat{x}}_{k}^{u}\left(1\right)$ and ${\hat{x}}_{k}^{u}\left(2\right)$ of DUCEKF (16) reproduced by DAC.	23	120
		TOTAL	68,300

From Figure A1, steps 1 through 8 are executed once and correspond to the initialization of the dsPIC, the TFT-LCD, and the DAC. Since these steps involve only hardware setup procedures, their execution times are excluded from the algorithm performance evaluation. Similarly, steps 20 to 22, which are responsible for generating the phase-plane plots and displaying the numerical values of the four state variables on the TFT-LCD, are also excluded from the analysis. Additionally, step 23, which involves outputting the temporary variables of systems (12) and (16) through DAC, is not considered in this evaluation. The performance evaluation focuses exclusively on the execution of the main algorithm, steps 9–19.

### Appendix A.1. Experimental Validation via DAC Output and TFT-LCD

The DAC60508, supporting 12-bit scaling, enables simultaneous output of the four state variables of the DHVPO (12) and DUCEKF (16) through synchronized updates. Data is pre-scaled to a positive range to ensure accurate representation, achieving precise reproduction in approximately 120 μs, which represents a short time duration essential for visualization in the time domain at the U2 output.

To graphically represent the phase-plane and the current state of the trajectories for systems DHVPO (12) and DUCEKF (16) on a TFT-LCD driven by the ILI9341 controller, the process begins in step 20 with the generation of point P1— consisting in depicting the states $x_{k}\left(1\right)$, $x_{k}\left(2\right)$, ${\hat{x}}_{k}^{u}\left(1\right)$, and ${\hat{x}}_{k}^{u}\left(2\right)$. This involves calculating four coordinates that are positively scaled to fit within the 320 × 240 pixel resolution. In step 21, point P2 is generated using the same procedure but with the states from the previous iteration, and in step 22, a line is drawn to connect P1 and P2, representing the dynamic trajectory on screen.

Text tools are simultaneously used to display numerically the four corresponding coordinates directly on the TFT-LCD. These values must be displayed, cleared, and updated for each new iteration to reflect the system’s evolving states. The complete graphical routine, covering steps 20 to 22, is executed in approximately 67.932 ms per cycle.

Given that the ILI9341 controller supports fast refresh rates—with typical write times under 1 ms for basic graphics—the available 68.3 ms per cycle provides ample time for all drawing and updating tasks. This ensures smooth, flicker-free, and real-time visualization of both the trajectories and their corresponding coordinate values on the TFT-LCD.

### Appendix A.2. Experimental Validation Using Execution Time-Paths

According to Figure A1, each execution route is assigned a distinct color for visual identification: Route 1 is red, Route 2 is blue, Route 3 is green, Route 4 is brown, Route 5 is orange, and Route 6 is purple. The main algorithm (steps 9–19) features six possible execution paths, determined by three conditional branches located at steps 10, 13, and 17.

- At step 10, an initial condition is evaluated. If the condition is true, the algorithm proceeds along Route 1 (0.2 μs); otherwise, it follows Route 2 (0.2 μs). The evaluation in this conditional aims to determine the previous DHVPO position to establish the direction of position change, whether decreasing or increasing, for the next conditional.
- step 13 evaluates a second condition. A true result leads to Route 3 (243 μs), while a false result directs execution to Route 4 (252 μs). This evaluation checks if the DHVPO has made contact with the impact surface. If the position is positive, falls within the activation set, and is decreasing, then it indicates that the impact surface has been reached.
- step 17 introduces a third conditional branch. If the condition holds, Route 5 (0.8 μs) is selected; otherwise, Route 6 (0.8 μs) is taken. This condition occurs only if the previous condition has not been met, which implies that the DHVPO did not hit the impact surface. In this case, it is necessary to verify whether the DUCEKF has impacted the surface. If it has, then the DUCEKF is positioned on the impact surface prior to the DHVPO, and a hold is applied since the DUCEKF is synchronized with the DHVPO to execute the transition. If the DUCEK has not hit the surface, the EKF continues its operation in the smooth branch.

These branching decisions determine both the timing and dynamic behavior of the main algorithm, enabling multiple execution paths depending on the logical conditions evaluated. A total of 350 iterations were performed, from k=1 to k=350, during which the following four execution paths were identified:

- Path 1: uses Routes 1, 4, and 6.
- Path 2: uses Routes 2, 4, and 6.
- Path 3: uses Routes 2 and 3.
- Path 4: uses Routes 2, 4, and 6.

By substituting the experimentally validated timing values of Routes 1–6, we present Table A2, which summarizes the four execution paths identified throughout the proposed 350 iterations.

**Table A2:** Execution paths and their corresponding routes.

Path	*k* Iteration	Route, Time (μs)	Route, Time (μs)	Route, Time (μs)	Qty
1	1	1, 0.2	4, 252	6, 0.8	1
2	2–314	2, 0.2	4, 252	6, 0.8	313
3	315	2, 0.2	3, 243	–	1
4	316–350	2, 0.2	4, 252	6, 0.8	35

### Appendix A.3. Statistical Analysis of Execution Times

We examine the total execution times associated with Paths 1 through 4, as detailed in Table A2, along with their respective occurrence counts. The following notation defines the parameters used to characterize the execution paths and their corresponding statistical metrics in the proposed main algorithm:

- *P*: total number of *k* executed iterations (i.e., 350 total iterations covering the occurrences of the four paths).
- $p_{i}$: number of occurrences for each unique execution path *i*, with i=1,2,3,4.
- $t_{i}$: total execution time for a complete route sequence corresponding to path *i*.

$$P=p_{1}+p_{2}+p_{3}+p_{4}$$

where:

$$p_{1}=1,\;p_{2}=313,\;p_{3}=1,\;p_{4}=35$$

⇒P=1+313+1+35=350

- Mean:
  The execution time for paths 1, 2, and 4 is identical and is denoted by $t_{1}$ = 253.0 μs. In contrast, path 3 has a different execution time, defined as $t_{2}$ = 243.2 μs. The sample weighted mean $\hat{m}$ is:
  $$\hat{m}=\frac{p_{1}t_{1}+p_{2}t_{1}+p_{3}t_{2}+p_{4}t_{1}}{P}=\frac{\left(p_{1}+p_{2}+p_{4}\right)t_{1}+p_{3}t_{2}}{P}$$
  $$\hat{m}=\frac{(1+313+35)\times 253.0+1\times 243.2}{350}=\frac{349\times 253.0+243.2}{350}=252.972\;\mu s$$
- Variance and Standard Deviation:
  The variance $\sigma^{2}$ is defined as:
  $$\sigma^{2}=\frac{p_{1}{\left(t_{1}-\hat{m}\right)}^{2}+p_{2}{\left(t_{1}-\hat{m}\right)}^{2}+p_{3}{\left(t_{2}-\hat{m}\right)}^{2}+p_{4}{\left(t_{1}-\hat{m}\right)}^{2}}{P}$$
  $$=\frac{\left(p_{1}+p_{2}+p_{4}\right){\left(t_{1}-\hat{m}\right)}^{2}+p_{3}{\left(t_{2}-\hat{m}\right)}^{2}}{P}$$
  Replacing with numbers:
  $$\sigma^{2}=\frac{349\cdot {\left(253.0-252.972\right)}^{2}+1\cdot {\left(243.2-252.972\right)}^{2}}{350}=\frac{349\cdot 0.000784+1\cdot 95.496}{350}$$
  $$=\frac{0.273616+95.496}{350}=\frac{95.7696}{350}=0.2736\;{\left(\mu s\right)}^{2}$$
  $$\sigma =\sqrt{\sigma^{2}}=\sqrt{0.2736}=0.5231\;\mu s$$

The mean execution time of approximately 252.972 μs represents the typical processing duration of the main algorithm running on the dsPIC under normal operating conditions. This average is predominantly influenced by the main execution paths (Paths 1, 2, and 4), which exhibit identical timing characteristics.

The remarkably low standard deviation of 0.5231 μs indicates outstanding temporal stability in the execution of the main algorithm. This consistency is crucial in embedded and real-time systems, where predictable timing guarantees system responsiveness and reliability.

This narrow variability around the mean suggests that external factors—such as interrupts, task switching, or clock variations—have minimal impact on processing time.

The presence of a single atypical execution path (Path 3), characterized by a faster execution time of 243.2 μs, represents a difference in the timing distribution. Although this outlier slightly lowers the overall mean, it does not substantially affect the overall timing consistency of the algorithm.
